# Unprecedented within-species chromosome number cline in the Wood White butterfly *Leptidea sinapis *and its significance for karyotype evolution and speciation

**DOI:** 10.1186/1471-2148-11-109

**Published:** 2011-04-20

**Authors:** Vladimir A Lukhtanov, Vlad Dincă, Gerard Talavera, Roger Vila

**Affiliations:** 1 Department of Karyosystematics, Zoological Institute of Russian Academy of Science, Universitetskaya nab. 1, 199034 St. Petersburg, Russia; 2 Department of Entomology, St. Petersburg State University, Universitetskaya nab. 7/9, 199034 St. Petersburg, Russia; 3 Institut de Biologia Evolutiva (CSIC-UPF), Passeig Marítim de la Barceloneta 37-49, 08003 Barcelona, Spain; 4 Departament de Genètica i Microbiologia, Universitat Autònoma de Barcelona, 08193 Bellaterra, Spain; 5 Institució Catalana de Recerca i Estudis Avançats (ICREA), Passeig Lluís Companys 23, 08010 Barcelona, Spain

## Abstract

**Background:**

Species generally have a fixed number of chromosomes in the cell nuclei while between-species differences are common and often pronounced. These differences could have evolved through multiple speciation events, each involving the fixation of a single chromosomal rearrangement. Alternatively, marked changes in the karyotype may be the consequence of within-species accumulation of multiple chromosomal fissions/fusions, resulting in highly polymorphic systems with the subsequent extinction of intermediate karyomorphs. Although this mechanism of chromosome number evolution is possible in theory, it has not been well documented.

**Results:**

We present the discovery of exceptional intraspecific variability in the karyotype of the widespread Eurasian butterfly *Leptidea sinapis*. We show that within this species the diploid chromosome number gradually decreases from 2n = 106 in Spain to 2n = 56 in eastern Kazakhstan, resulting in a 6000 km-wide cline that originated recently (8,500 to 31,000 years ago). Remarkably, intrapopulational chromosome number polymorphism exists, the chromosome number range overlaps between some populations separated by hundreds of kilometers, and chromosomal heterozygotes are abundant. We demonstrate that this karyotypic variability is intraspecific because in *L. sinapis *a broad geographical distribution is coupled with a homogenous morphological and genetic structure.

**Conclusions:**

The discovered system represents the first clearly documented case of explosive chromosome number evolution through intraspecific and intrapopulation accumulation of multiple chromosomal changes. *Leptidea sinapis *may be used as a model system for studying speciation by means of chromosomally-based suppressed recombination mechanisms, as well as clinal speciation, a process that is theoretically possible but difficult to document. The discovered cline seems to represent a narrow time-window of the very first steps of species formation linked to multiple chromosomal changes that have occurred explosively. This case offers a rare opportunity to study this process before drift, dispersal, selection, extinction and speciation erase the traces of microevolutionary events and just leave the final picture of a pronounced interspecific chromosomal difference.

## Background

Despite the fundamental role of chromosomal change in eukaryotic evolution, the mechanisms related to this process are still poorly known. Main karyotypic features of organisms, such as the number of chromosomes, are usually stable within species [1,2]. This stability is in good correspondence with the fact that new chromosomal rearrangements usually originate as heterozygotes and are often - although not always - associated with heterozygote disadvantage (=negative heterosis; =underdominance). Therefore, their spread to fixation within a large population has low probability [2]. At the same time, differences in karyotype characters between species, including diploid chromosome number (2n), are extremely common. Numerous cases of extraordinary differences in chromosome number, especially in plants, are due to polyploidy [3]. Even when excluding polyploidy, interspecific variation remains very frequent, and many closely related species often have substantially different chromosome numbers. In metazoan animals, the greatest range of within-genus karyotype variation not related to polyploidy is found in *Agrodiaetus *blue butterflies, where diploid chromosome number ranges between species from 2n = 20 to 2n = 268 in spite of morphological similarity and very recent time of species divergence [4]. Interestingly, *Agrodiaetus *also tends to demonstrate the greatest karyotype difference between very closely related species, e.g. sister species *A. biruni *and *A. posthumus *have 2n = 20 and 2n = 180 respectively with no intermediates between them.

In vertebrates, the range of chromosome number variation between closely related species is smaller, yet still impressive. For example, the analysis of 11 species of the catfish genus *Corydoras *revealed that they have karyotypes ranging from 2n = 44 to 2n = 102 [5]. The tuco-tucos, South American rodents of the genus *Ctenomys*, show chromosomal variation with diploid numbers varying from 2n = 10 to 2n = 70 among the 60 species described [6]. The deer genus *Muntiacus *includes species with different karyotypes, ranging from 2n = 6 to 2n = 46 [7]. In plants, the greatest range of within-genus karyotype variation not related to polyploidy is found in *Carex*, where diploid chromosome number ranges from 2n = 12 to 2n = 132 [8].

The discrepancy between intra- and interspecific variability in chromosome numbers poses a serious evolutionary problem. How can numerous species with extremely diverse karyotypes evolve in a relatively short period of time, if major chromosomal rearrangements changing the number of chromosomes are mostly underdominant and, consequently, intraspecific variations are rare and their range is limited?

One possible explanation is that extremely different chromosome numbers evolve gradually through multiple speciation/raciation events, each involving the fixation of a single (or few) chromosomal rearrangement(s), and followed by the subsequent extinction of species or races with intermediate karyotypes. This step-by-step mechanism of karyotype evolution seems to be common in nature, and its initial phase can be observed in some chromosomally polymorphic organisms such as the mouse *Mus musculus domesticus *and the shrew *Sorex araneus *[9-13]. It has been recently demonstrated that the reduction in fertility of hybrids between the house mouse races separated by fixed monobrachial differences is not so pronounced as previously supposed [14]. Nevertheless, this study generally supported the chromosomally-based monobrachial speciation model as a process that accelerates the acquisition of reproductive isolation in the house mouse [14]. In the step-by-step process, the transitional forms are expected to demonstrate a chromosomal fusion/fission polymorphism and, accordingly, numerous examples are known where single or few chromosomal fusions exist in the polymorphic phase, e.g., Robertsonian fusions in *Drosophila americana *[15], melanopline grasshoppers [16] and rodents of the genus *Ctenomys *[6,17].

An alternative hypothesis is that dramatic changes in chromosome number appear as a consequence of a within-species accumulation of numerous chromosomal rearrangements, resulting in highly polymorphic systems with the subsequent extinction of intermediate karyomorphs. A necessary precondition for this mechanism is that major chromosomal rearrangements changing the number of chromosomes are not strongly underdominant. This seems to hold true for different groups such as butterflies, flies, grasshoppers, spiders, fishes and mammals [6,15-25].

While the within-species mechanism of explosive chromosome number evolution is possible in theory, it has been less well documented compared to the evolution through multiple speciation/raciation events. In practice, it is difficult to record such an extensive within-species accumulation for two reasons. First, the transition from one chromosomal form to another may be very fast compared to the species lifespan. The only exception is the chromosomal evolution operated by balancing selection. However, this mechanism seems to be rare, except in the case of inversions [[26,27], but see [28]]. Second, even if polymorphism for multiple chromosomal rearrangements is found, it may be difficult to distinguish between a polymorphic system primarily evolved within a species and a polymorphism resulting from hybridization between different, chromosomally diverged species. For example, in the hybridization zones between low and high chromosome number species of the rodent genus *Ellobius*, there is a so-called "chromosomal fan" including all chromosome numbers from 2n = 31 to 2n = 54 [29]. In fact, this case does not represent evidence for within-species accumulation of chromosomal changes, but simply represents the outcome of secondary parapatry by previously isolated chromosomal races.

Furthermore, the clinal geographical distribution of chromosomal races observed in some organisms [1,2], apparently compatible with gradual within-species accumulation of chromosomal changes, may be better explained by the multiple speciation mechanism. For example, in butterflies of the *Erebia tyndarus *complex there are several geographically isolate chromosomal races (chromosome numbers ranging from 2n = 16 to 2n = 102) [30], and in fossorial mole rats of the *Spalax ehrenbergi *complex four linearly distributed chromosomal races exist (from 2n = 52 to 2n = 60) [31]. In these cases, intrapopulation chromosomal polymorphism is absent and differences between neighbouring chromosomal races, although minor, are fixed. Detailed molecular and morphological studies provide evidence for non-conspecificity of the *E. tyndarus *and *S. ehrenbergi *forms, and several distinct species were identified and formally described [32,33].

In this study we describe a chromosomal cline in the Wood White butterfly, *Leptidea sinapis *(Insecta, Lepidoptera, Pieridae) that provides strong evidence for rapid and extensive within-species chromosome number evolution through accumulation of multiple chromosomal changes. This cline is exceptional in the geographic area that it covers (6000 km) and in its range of within-species chromosome number variation (2n = 56-106). Excluding polyploidy, this is the widest known within-species chromosome number range for any animal or plant, and it is comparable with the highest known level of within-genus karyotype variability.

## Results and Discussion

We analyzed the karyotype, mitochondrial and nuclear genetic markers, and the morphology of the Wood White butterfly *L. sinapis*. This is a common species widely distributed from Portugal and Spain in the west to Siberia in the east [34]. From this territory different chromosome numbers have been reported in literature ranging from n = 28 to n = 41 [35]. However, these results are impossible to interpret in practice because of the discovery in 1993 of a cryptic sympatric species (*L. reali*) in Europe and Asia [36]. As all karyotype data for *L. sinapis *were published before this date, it is unclear whether reported chromosome numbers reflect inter- or intraspecific variability.

**Figure 1:**
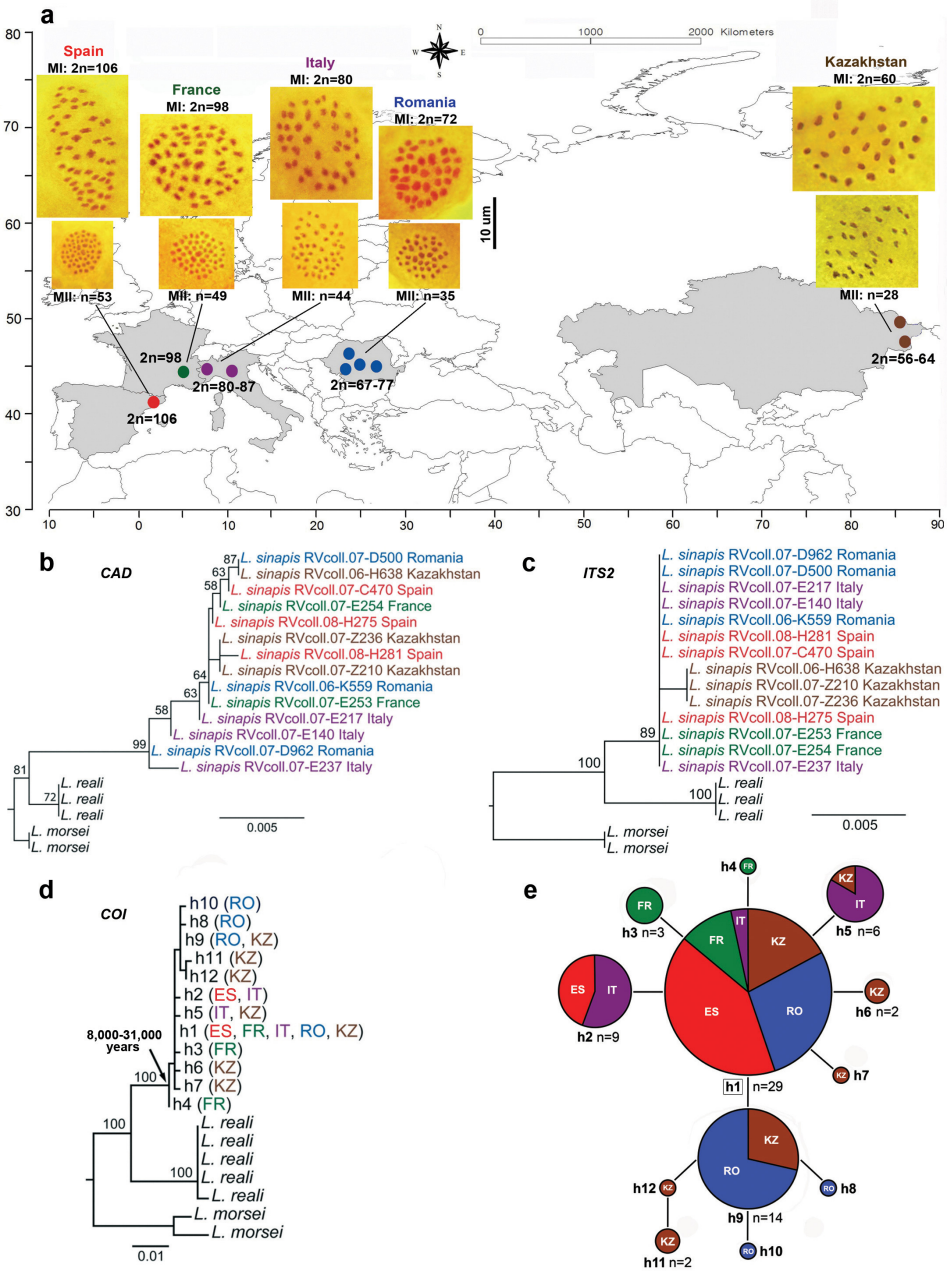
**Chromosomal cline in *Leptidea sinapis *across the Palaearctic region**. **a**. Sampling sites and karyotype results. Metaphase plates were observed in 35 individuals, out of which 23 had unambiguous chromosome number counts: Spain - 4, France - 2, Italy - 2, Romania - 8, Kazakhstan - 7. Top row of microphotographs: examples of diploid chromosome number (2n) counted in metaphase I of meiosis (MI). Bottom row of microphotographs: examples of haploid chromosome number (n) counted in metaphase II of meiosis (MII). Maximum likelihood trees for **b**. *CAD*, **c**. *ITS2 *and **d**. *COI*. Bootstrap supports (>50%) are shown for each node. **e**. Most parsimonious *COI *haplotype network. Colours refer to each studied region as indicated in the map. ES - Spain, FR - France, IT - Italy, RO - Romania, KZ - Kazakhstan.

**Figure 2:**
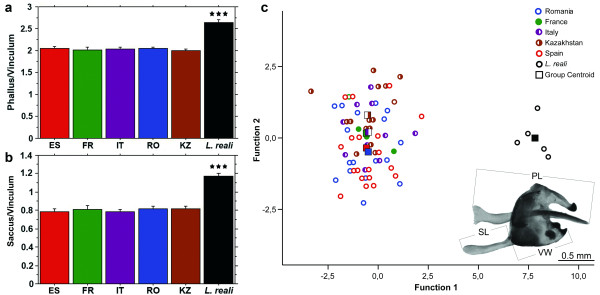
**Male genitalia morphology of *Leptidea sinapis *reveals no significant intraspecific differences**. One-way ANOVA for **a**. phallus length/vinculum width and **b**. saccus length/vinculum width. The sibling species *L. reali *is included as positive control. Only *L. reali *versus all *L. sinapis *groups is significantly different (p < 0.0001 for both analyses). The bars represent two standard errors. **c**. Canonical discriminant analysis based on phallus length, saccus length and vinculum width.

Our study covers populations from different parts of the *L. sinapis *distribution (Figures 1, 2), as well as the closely related species *L. reali *and *L. morsei *as comparison. We discovered that diploid chromosome number ranges in *L. sinapis *from 2n = 106 in Spain to 2n = 56 in eastern Kazakhstan in a longitudinal cline (Figure 1a; for more details, see Additional file 1). These findings are based on the examination of 209 male specimens, with metaphase plates observed in 35 individuals, out of which 23 had unambiguous chromosome number counts (Spain - 4, France - 2, Italy - 2, Romania - 8, Kazakhstan - 7). We also found that chromosome numbers are not stable within some populations from Italy, Romania and Kazakhstan. Specimens with different chromosome numbers were found within each of these populations, and the great majority of the individuals were chromosomal heterozygotes displaying from one to six multivalents in metaphase I of meiosis (Additional file 1, Figure S1). In the heterozygotes, we observed no abnormalities in the anaphase I stage of meiosis, and the first division of meiosis resulted in normal haploid metaphase II cells where, as expected, two types of metaphase plates with different chromosome numbers were observed. Therefore we conclude that chromosomal rearrangements are not fixed in several of the populations studied, and there seems to be no strong selection against chromosomal heterozygotes. Interestingly, chromosome number range overlaps between some studied populations separated by hundreds of kilometers, e.g. in Kazakhstan between the population from Landman (2n = 56-61) and the population from Saur (2n = 56-64).

In certain species, variation in chromosome number may be caused by the presence of so-called B-chromosomes (=additional chromosomes, =supernumerary chromosomes) [37]. B-chromosomes consist mainly of repetitive DNA and can be usually found in low numbers (one to five) in a percentage of the individuals of a given population. Although they are dispensable, they can sometimes accumulate through processes of mitotic or meiotic drive [38]. B-chromosomes can be distinguished from normal A-chromosomes because they are usually smaller and can be seen as additional chromosomes present in only some of the individuals in a population. The best diagnostic feature is their identity at meiosis, where they may be found as univalents, or in various pairing configurations (bivalents or multivalents), but never pairing with A-chromosomes. Thus, meiotic analysis is critical to distinguish between B-chromosomes and normal A-chromosomes [37,38]. Although we cannot totally exclude that B-chromosomes can be found in *L. sinapis*, especially taking into account that they are known in other genera of the family Pieridae [39], there is good evidence that B-chromosomes are not a valid explanation for the chromosome number cline found in *L. sinapis*. This is due to the fact that in the Spanish population, where the number of chromosomes is maximal (and correspondingly where the highest number of B-chromosomes would be expected), they seem to be completely absent: the chromosome number is stable within as well as between individuals, and no univalents have been observed during meiosis. Moreover, no univalents have been observed during meiosis in any of the other populations studied. Additionally, the following clear pattern was observed: the higher the chromosome numbers in a population, the smaller the size of chromosomes, and vice versa (Figure 1; Additional file 1, Figure S1). This regularity indicates that chromosomal fusions/fissions (but not B-chromosomes) were the main mechanism of karyotype evolution.

*Leptidea sinapis *can be distinguished from its closest relative *L. reali *by the length of the phallus, saccus and vinculum (in male genitalia) or of the ductus bursae (in female genitalia) [36,40] as well as by molecular markers [41,42]. Therefore, to exclude the possibility of cryptic species involved in the formation of the extraordinarily high chromosomal variability and to demonstrate the conspecificity of the populations studied, we performed morphological and molecular analysis of each studied individual.

The measured variables of the male genitalia showed no significant difference or apparent trend between chromosomal races according to one-way ANOVA (Figure 2a, b) and to discriminant analysis (DA) (Figure 2c). 100% of the *L. reali *were correctly classified to species with the DA, but within *L. sinapis*, between 0 (France and Italy) and 62.5% (Kazakhstan) of specimens were correctly assigned to region (Additional file 1, Table S1).

**Figure 3:**
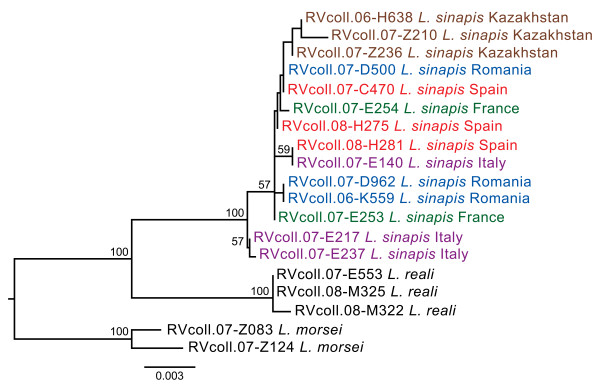
**Maximum Likelihood tree of *Leptidea sinapis *based on the combined analysis of mitochondrial *COI *and nuclear *CAD *and *ITS2 *according to the HKY model (log likelihood score = -3159.19036) and 100 bootstrap replicates**. The scale bar represents 0.003 substitutions/position.

The mitochondrial *Cytochrome Oxidase I *(*COI*) and nuclear *carbamoyl-phosphate synthetase 2/aspartate transcarbamylase/dihydroorotase *(*CAD*) and *internal transcribed spacer 2 *(*ITS2*) markers analyzed did not reveal deep intraspecific levels of divergence (maximum uncorrected p distance of 0.61% for *COI*, 0.7% for *CAD *and 0.16% for *ITS2*) suggesting the absence of cryptic species (Figures 1b-d and Figure 3). The *COI *haplotype network (Figure 1e) shows that the maximum connection steps are only four, and that the most common haplotype is found in all the studied regions. The observed genetic variability is rather low for an almost pan-Palaearctic species (e.g. [42,43]), even more so since *L. sinapis *is considered a non-migratory poor flyer. The fact that the same low variability is shown by several independent markers rejects a recent mitochondrial genetic sweep and strongly suggests a very recent geographic expansion. Coalescence-based dating with each marker and with all the markers combined estimates that the time to the most recent common ancestor of all the populations is only 8,500 to 31,000 years. Thus, we conclude that there is no evidence for multiple species involved in the formation of the discovered cline, and that its origin is very recent.

It is known that in some systems, variation in chromosome number may be a result of ongoing hybridization between different, chromosomally diverged species [29]. Therefore, the chromosome number variability discovered may be a consequence of hybridization between *L. sinapis *and its sibling species *L. reali*. This explanation may seem possible given that the presence of putative F_1 _hybrids between *L. sinapis *and *L. reali *was suggested [44]. However, these results [44] were based on some apparent mismatches between DNA-based identifications (which were congruent for RAPD markers and *COI*) and morphometry of the male genitalia. The classification of the sequenced specimens based on their genitalia was made by employing a bivariate plot, which took into account only the lengths of the phallus and saccus. A recent comprehensive morphometrical study on *L. sinapis *and *L. reali *from Central Italy [40] highlighted the limitation of the "phallus and saccus" approach, which can lead to ambiguous classifications. The same study showed that this limitation can be corrected when using additional genitalic characters (especially the vinculum width) and performing multivariate analyses. Therefore, the report of possible hybrids between *L. reali *and *L. sinapis *requires confirmation since it may actually represent an artifact caused by the interpretation of insufficient morphological traits. Moreover, in case of interspecific hybridization we can expect that some individuals would be heterozygous for species-specific nuclear molecular markers and specimens with intermediate morphology of genitalia should be found. None of the specimens studied in our work has shown these characteristics (see above). Due to genitalic morphological constraints between the two species, introgression is likely to be unidirectional with female *L. sinapis *potentially inseminated by male *L. reali *[36,44]. Finally, several studies dealing with the mating behaviour of *L. sinapis *and *L. reali *reported that females of both species exclusively mated with conspecific males, suggesting the presence of strong precopulatory barriers [36,45,46]. Therefore, we can conclude that interspecific hybridization is an unlikely explanation for the origin of the discovered chromosomal cline.

**Figure 4:**
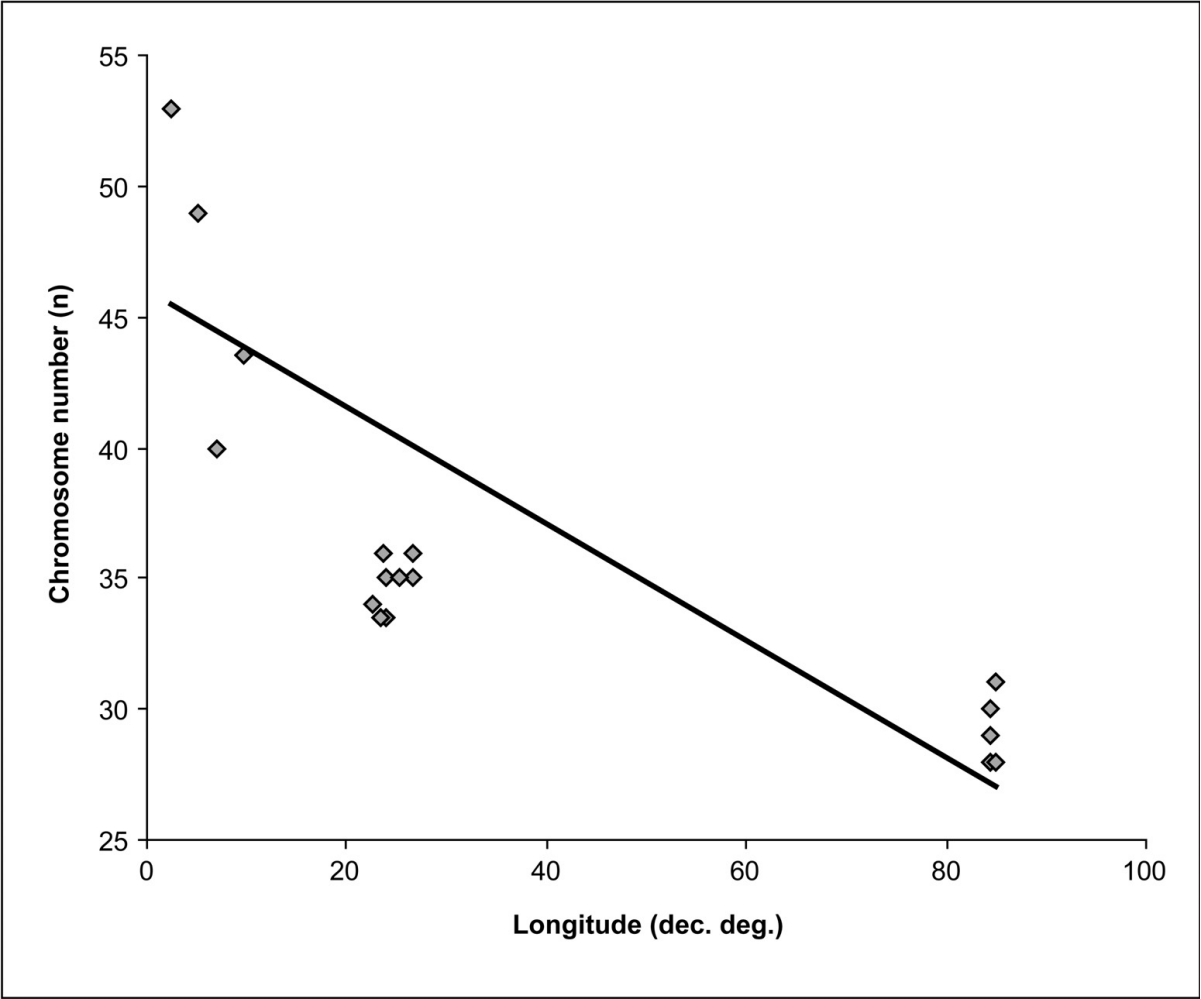
**Variation of *L. sinapis *chromosome number across geographical longitude**. Chromosome number is inversely correlated with longitude according to a linear function (r = 0.826; p < 0.0001). Results based on 23 specimens with unambiguous chromosome number counts (Spain - 4, France - 2, Italy - 2, Romania - 8, Kazakhstan - 7).

The clinal distribution of chromosome numbers in *L. sinapis *is statistically significant (p < 0.0001) and it is very unlikely to have arisen by chance (Figure 4). Interestingly, the cline is longitudinally oriented (Figure 1a), indicating either the direction of selective pressure involved in its formation, or the direction of population dispersal, or both of these processes. According to our dating, the moment of this dispersal would correspond to the upper Pleistocene and the Holocene, a period characterized by a strong glaciation in northern Europe and the Alps [47]. Thus, our estimates indicate that the dispersal of *L. sinapis *could have occurred before or after the last glacial maximum (24,000 to 17,000 years ago).

Several other cases of broad intraspecific chromosomal polymorphism have been described in animals [6,18-21,23,24,48-56] and plants [8,57]. However, all these cases differ from the cline found in *L. sinapis *by the essentially smaller range of karyotype variability and by the possible existence of two or more cryptic species involved in the formation of the polymorphic chromosomal system. In order to demonstrate the intraspecific nature of karyotype variability, the following three criteria should be met simultaneously: 1) segregating chromosomal polymorphism within a population should be demonstrated, 2) molecular markers should not suggest the presence of potential cryptic species, and 3) species-diagnostic morphological differences should be lacking. To our knowledge, only studies on the common shrew and the house mouse have met all these criteria, but chromosomal races within these mammals have essentially smaller differences in chromosome number and apparently evolved through a step-by-step accumulation of single chromosomal rearrangements [9-13] rather than through wide intraspecific and intrapopulation chromosome number polymorphism.

## Conclusions

Given that (a) chromosomal races of *L. sinapis *belong to the same species, (b) intrapopulation chromosome number polymorphism exists, (c) the chromosome number range overlaps between some populations separated by hundreds of kilometers, (d) the species has broad ecological preferences and is widely distributed, (e) the species has a rather homogenous genetic structure, and (f) chromosomal heterozygotes are abundant, this represents a clearly documented case of rapid and massive within-species accumulation of multiple chromosomal rearrangements affecting the number of chromosomes.

The chromosomal rearrangements discovered in our investigation display segregating polymorphism that seems not to strongly affect reproductive fitness within the populations studied. However, these rearrangements are not necessarily irrelevant to the process of formation of reproductive isolation (i.e. to speciation). It is well known that Robertsonian rearrangements (i.e. nonreciprocal translocations involving fission and fusion at or near a centromere), have the potential to limit gene flow and drive speciation [58,59]. The Wood White butterfly, like other Lepidoptera and some other insects, has holokinetic chromosomes in which the centromere is not localized and centromeric activity is distributed along the length of the chromosome [35,60-62]. It has been recently demonstrated that fusions/fissions of holokinetic chromosomes restrict gene flow too, and that this effect is cumulative (i.e. increases proportionally with the level of chromosomal differences) [57]. In the case of *L. sinapis *all evidence suggests that neighbour populations with relatively low differences in chromosome number are reproductively compatible. We cannot exclude that geographically distant and chromosomally divergent populations would display reduced fertility if crossed, although they are connected by a chain of compatible populations that should allow gene flow. Therefore, the discovered system opens the possibility to study clinal speciation, a process that is theoretically possible but difficult to document [[63], pages 113-123].

Chromosomal rearrangements are known to limit introgression in parapatry or sympatry with regard to isolation genes, thus facilitating the maintenance of incipient species boundaries [64,65], and serving as regions where isolation genes can accumulate [15,27,66-68]. The preservation and/or accumulation of isolation genes protected by chromosomal rearrangements could represent a prerequisite for speciation by means of suppressed-recombination mechanisms [15,27,64-68].

In conclusion, the *L. sinapis *chromosomal cline seems to represent a narrow time-window of the very first steps of species formation linked to multiple chromosomal changes that have occurred explosively. This case offers a rare opportunity to study this process before drift, dispersal, selection, extinction and speciation erase the traces of microevolutionary events and just leave the final picture of a pronounced interspecific chromosomal difference.

## Methods

Note: During the publication process of this paper it has been shown that the Romanian specimens of *L. reali *used here as outgroup actually belong to a new cryptic species named *Leptidea juvernica *[69].

### Sample collecting

Fresh male *Leptidea *specimens (Additional file 1, Table S2) were collected with the insect net and were kept alive in glassine envelopes. In the laboratory, butterflies were killed by pressing the thorax and testes were removed from the abdomen and immediately placed into a 0.5 ml vial with freshly prepared Carnoy fixative (ethanol and glacial acetic acid, 3:1). Bodies were placed into a 2 ml plastic vial with 100% ethanol for DNA analysis and wings were stored in glassine envelopes. Each sample has been assigned a unique sample ID. All the samples are stored in Roger Vila's DNA and Tissues Collection in Barcelona, Spain.

### Genitalia preparation and morphometric analyses

Male genitalia were prepared according to the following protocol: maceration for 15 minutes at 95°C in 10% potassium hydroxide, dissection and cleaning under a stereomicroscope and storage in tubes with glycerin. Genitalia were photographed laterally (Figure 2c), without being pressed, in a thin layer of distilled water under a Carl Zeiss Stemi 2000-C stereomicroscope equipped with a DeltaPix Invenio 3S digital camera. Measurements were performed based on the digital photographs by using AxioVision software. A total of 73 specimens of *L. sinapis *were included in the morphometrical analyses (Additional file 1, Table S3). These included 35 of the karyotyped samples, and 38 individuals collected in the same locality and moment for which the cytogenetic studies did not produce results. In addition, five specimens of the sibling *L. reali *were added as outgroup. Three elements of the male genitalia were measured: phallus, saccus and vinculum width. These are the best diagnostic characters to separate *L. sinapis *from *L. reali *[40]. The vinculum width was used to normalize the size of the specimen.

StatView 5.0.1 (SAS Institute Inc., 1992-1998) was used to perform one-way ANOVA in order to test for differences in the length of the phallus and saccus, each normalized by the width of the vinculum, between regions for *L. sinapis*, and between *L. sinapis *and *L. reali*. All variables were normally distributed (Kolmogorov-Smirnov Test, p > 0.05). The software SPSS 14.0 was used to perform a discriminant analysis by employing the stepwise method. The Box's M test was used to evaluate the homogeneity of covariance assumption (p > 0.05). The variables were selected with the Wilks' lambda statistic, which measures how each function separates cases into groups. In order to test the obtained classification a cross validation was carried out.

### Karyotype analyses

Gonads were stored in Carnoy fixative (ethanol and glacial acetic acid, 3:1) for 2-6 months at 4ï‚°C and then stained with 2% acetic orcein for 30 days at 20ï‚°C as it was previously described [70,71].

Chromosomes of butterflies (Lepidoptera) are small, numerous and uniform in both shape and size [35]. They lack a distinct primary constriction (the centromere) and are regarded as holokinetic with kinetochores extended over a large portion of the chromosome surface [60]. The uniformity of lepidopteran chromosomes, the absence of morphological markers such as the centromeres and the lack of convenient differential banding techniques [61] make difficult the identification of individual chromosomes by standard cytogenetic methods. Although new approaches to individual identification of the Lepidoptera chromosomes based on the fluorescent *in situ *hybridization (FISH) technique have been recently elaborated [72-74], they are applicable only for studying species bred in the laboratory. For this reason, the chromosome number remains the most commonly used karyotypic character in Lepidoptera cytogenetics and karyosystematics. In our study we counted the diploid chromosome numbers (2n) in mitotic spermatogonial cells and the haploid chromosome numbers (n) in metaphase II of male meiosis. We also counted the number of chromosomal elements (n) (bivalents + multivalents) in metaphase I of male meiosis. In the last case, the number of chromosomal elements was equal to the haploid number (n) if all the elements were represented by bivalents, or less if some elements were represented by multivalents. To distinguish between bivalents and multivalents, we used a special method [75]. Briefly, by varying the pressure on the coverslip, we were able to manipulate chromosomes, e.g. change their position and orientation in intact (not squashed) spermatocyte cells, and consequently to analyze the structure of the bivalents and multivalents.

In total, preparations from 209 males were analyzed. As cell divisions are extremely rare in *Leptidea *during imago stage [76], metaphase plates were observed in only 35 individuals (Additional file 1, Table S2). These individuals were also used for morphological and molecular analysis.

### Geographical longitude vs. chromosome number

Pearson correlation coefficients were used to assess the degree of association between haploid karyotype and geographical longitude. Longitude was measured in decimal degrees and only 23 samples with unambiguous chromosome number counts were included (see Additional file 1, Table S4). If the specimen showed different chromosome numbers in different cells, the average between the different chromosome numbers was used.

### Specimen sequencing

Total genomic DNA was extracted using Chelex 100 resin, 100-200 mesh, sodium form (Biorad), under the following protocol: one leg was removed and introduced into 100 μl of Chelex 10% and 5 μl of Proteinase K (20 mg/ml) were added. The samples were incubated overnight at 55°C, afterwards were incubated at 100°C for 15 minutes and were subsequently centrifuged for 10 seconds at 3000 rpm.

A 676 bp fragment at the 5' end of the mitochondrial gene cytochrome oxidase subunit I (*COI*) was amplified by polymerase chain reaction using the primers LCO 1490 (5'-GGTCAACAAATCATAAAGATATTGG-3') [77] and Nancy (5'-CCCGGTAAAATTAAAATATAAACTTC-3') [78]. When these primers failed, we used the primers LepF1 (5'-ATTCAACCAATCATAAAGATATTGG-3') and LepR1 (5'-TAAACTTCTGGATGTCCAAAAAATCA-3') [79], which amplified a 658 bp fragment of *COI*. Double-stranded DNA was amplified in 25 μl volume reactions: 13.22 μl ultra pure (HPLC quality) water, 2.5 μl 10× buffer, 4.5 μl 25 mM MgCl2, 0.25 μl 100 mM dNTP, 1.2 μl of each primer (10 mM), 0.13 μl *Taq *DNA Gold Polymerase (Qiagen) and 2 μl of extracted DNA. The typical thermal cycling profile was: 95°C for 60 seconds, 44°C for 60 seconds and 72°C for 90 seconds, for 40 cycles. A total of 70 *L. sinapis *samples were successfully sequenced for this marker. These included 34 of the karyotyped samples, and 36 individuals collected in the same locality as the karyotyped samples. Five *L. reali *and two *L. morsei *specimens were also sequenced and used as outgroup.

A 571 bp fragment at the 5' end of the nuclear gene *CAD *was amplified by polymerase chain reaction using the primers CADFa (5'-GDATGGTYGATGAAAATGTTAA-3') and CADRa (5'- CTCATRTCGTAATCYGTRCT-3') (designed by A. Kaliszewska). Double-stranded DNA was amplified in 25 μl volume reactions: 16.65 μl ultra pure (HPLC quality) water, 2.5 μl 10× buffer, 1 μl 100 mM MgCl2, 0.25 μl 100 mM dNTP, 1.2 μl of each primer (10 mM), 0.2 μl *Taq *DNA Polymerase (Bioron, GmbH) and 2 μl of extracted DNA. The typical thermal cycling profile was: 95°C for 60 seconds, 48°C for 60 seconds and 72°C for 90 seconds, for 40 cycles. A total of 14 samples (all karyotyped) were sequenced for this marker. Three *L. reali *and two *L. morsei *specimens were also sequenced and used as outgroup.

A 684 bp fragment at the 5' end of the nuclear internal transcribed spacer 2 (*ITS2*) was amplified by polymerase chain reaction using the primers ITS3 (5'-GCATCGATGAAGAACGCAGC-3') and ITS4 (5'-TCCTCCGCTTATTGATATGC-3') [80]. Double-stranded DNA was amplified in 25 μl volume reactions: 16.7 μl ultra pure (HPLC quality) water, 2.5 μl 10× buffer, 1 μl 100 mM MgCl2, 0.25 μl 100 mM dNTP, 1.2 μl of each primer (10 mM), 0.15 μl *Taq *DNA Polymerase (Bioron, GmbH) and 2 μl of extracted DNA. The typical thermal cycling profile was: 95°C for 45 seconds, 47°C for 60 seconds and 72°C for 60 seconds, for 40 cycles. A total of 14 samples (all karyotyped) were sequenced for this marker. Three *L. reali *and two *L. morsei *specimens were also sequenced and used as outgroup. PCR products were purified and sequenced by Macrogen Inc. (Seoul, Korea). Sequences obtained specifically for this study were deposited in GenBank (accession numbers indicated in Additional file 1, Table S2).

### Sequence alignment and phylogenetic inference

*COI*, *ITS2 *and *CAD *sequences were edited and aligned using Geneious Pro 4.7.5 [81]. These resulted in three final alignments of 658 bp and 77 specimens for *COI*, 571 bp and 19 specimens for *CAD*, and 684 bp and 19 specimens for *ITS2*. For *COI*, duplicate haplotypes were removed using Collapse 1.2 [82]. Maximum Likelihood (ML) phylogenetic trees were inferred for *CAD*, *ITS2 *and *COI *using Phyml 2.4.4 [83], with the nucleotide substitution model HKY [84] for nuclear markers and HKY+I for *COI*, as suggested by jModeltest 0.1 [85], and 100 bootstrap replicates.

### Haplotype network

In order to examine relationships among haplotypes, a maximum parsimony haplotype network was constructed using TCS 1.21 [86]. The haplotype network was built with a 99% parsimony connection limit. The network presented one loop, which was broken according to frequency and geographic criteria [87].

### Estimation of TMRCA

Time to the most recent common ancestor (TMRCA) of *L. sinapis *was inferred with BEAST v.1.5.3 [88] independently for *COI*, *ITS2 *and *CAD *haplotypes under a Coalescent model with constant population size. Duplicate haplotypes were removed from the matrix using Collapse 1.2 [82]. A lognormal distribution (Mean = 0.15, Stdev = 0.798) was used assuming a maximum possible limit of 405000 years as the 95% HPD of the distribution, trying to let the maximum exploratory space to MCMC runs. To estimate this prior, we used the maximum *COI *intraspecific divergence for *L. sinapis *under a rather slow invertebrate mitochondrial substitution rate: 1.5% uncorrected pairwise distance per million years [89]. Since substitution rates are known to overestimate ages for recent lineages still under the coalescence process, we are certain that 405000 years is a good maximum estimate for the TMRCA of this species. The dataset was analyzed using the HKY model and applying a strict molecular clock along the branches. Base frequencies were estimated and a randomly generated initial tree was used. Parameters were estimated using two independent runs of 10 million generations each (with a pre-run burn-in of 100,000 generations) to ensure convergence, checked with the program Tracer v1.4.

A multi-locus approach with *BEAST [90] was also employed to check the results with a smaller set of 12 samples, including those with most divergent *COI *haplotypes. In order to study the effect of the outgroup, *COI *and multilocus analyses were conducted by both including and excluding *L. reali *haplotypes (Additional file 1, Table S5).

## Authors' contributions

VAL, VD, GT and RV designed the experiments and analyzed the data, VAL performed the analysis of karyotypes, VD and GT performed phylogenetic analyses, VD performed morphological analyses, VAL, VD and RV wrote the paper, and all co-authors contributed in the form of discussion and suggestions. All authors read and approved the final manuscript.

## Supplementary Material

Additional file 1 **Additional Text, Figures and Tables**. a) Additional results of chromosomal analyses. b) Figure S1. Karyotypes of *Leptidea sinapis*. c) Table S1. Discriminant analysis classification results for chromosomal races of *L. sinapis *and *L. reali*. d) Table S2. List of specimens included in this study. e) Table S3. Results of morphometric analysis of the male genitalia. f) Table S4. List of the specimens included in the analysis of geographical longitude vs. chromosome number. g) Table S5. Estimation of TMRCA of *L. sinapis *under a coalescent model. Click here for file
