# Supplementary material for: Unprecedented within-species chromosome number cline in the Wood White butterfly Leptidea sinapis and its significance for karyotype evolution and speciation
Source: BMC Evol Biol. 2011 Apr 20;11:109. doi: 10.1186/1471-2148-11-109 (PMC3113740; doi:10.1186/1471-2148-11-109)
Supplement: Additional file 1 — Additional Text, Figures and Tables. a) Additional results of chromosomal analyses. b) Figure S1. Karyotypes of Leptidea sinapis. c) Table S1. Discriminant analysis classification results for chromosomal races of L. sinapis and L. reali. d) Table S2. List of specimens included in this study. e) Table S3. Results of morphometric analysis of the male genitalia. f) Table S4. List of the specimens included in the analysis of geographical longitude vs. chromosome number. g) Table S5. Estimation of TMRCA of L. sinapis under a coalescent model. [file 1471-2148-11-109-S1.PDF]

Additional file

Unprecedented within-species chromosome number cline in the Wood White butterfly *Leptidea sinapis* and its significance for karyotype evolution and speciation

Vladimir A. Lukhtanov, Vlad Dincă, Gerard Talavera & Roger Vila

Additional chromosomal analyses results (fig. S1)

Spain

L. sinapis (RVcoll.08-H275) 2n= 106[1].Only mitotic cell divisions were found in this specimen demonstrating 106 chromosomes in diploid set.

L. sinapis (RVcoll.08-H281) 2n= 106. Only mitotic cell divisions were found in this specimen demonstrating 106 chromosomes in diploid set.

L. sinapis (RVcoll.07-C470) [2n=106][2]. Only MII cells were found in this specimen with all the metaphase plates demonstrating 53 chromosomes. The diploid number can be reconstructed as 2n=106.

L. sinapis (RVcoll.07-F568) [2n=106]. Only MI cells were found in this specimen with all the metaphase plates demonstrating 53 bivalents. The diploid number can be reconstructed as 2n=106.

France

L. sinapis (RVcoll.07-E253) [2n=98]. Only MI cells were found in this specimen with all the metaphase plates demonstrating 49 bivalents. The diploid number can be reconstructed as 2n=98.

L. sinapis (RVcoll.07-E254) [2n=98]. Only MII cells were found in this specimen with all the metaphase plates demonstrating 49 chromosomes. The diploid number can be reconstructed as 2n=98.

Italy

L. sinapis (RVcoll.07-E140) [2n=87]. This specimen was heterozygous for one chromosomal fusion/fission. In MI stage 42 bivalents and 1 trivalent were observed. In MII stage the metaphase plates with n=43 and with n=44 were observed. Accordingly, the diploid number can be reconstructed as 2n=87.

L. sinapis (RVcoll.07-E217) [2n=ca. 84-86]. Only MI cells were found. The number of bivalents was counted with an approximation as ca. 42-43. Accordingly, the diploid number can be reconstructed with an approximation as 2n=ca. 84-86.

L. sinapis (RVcoll.07-E237) [2n=80]. In MI cells all the metaphase plates demonstrated 40 bivalents. In MII cells all the metaphase plates demonstrated 40 chromosomes. Accordingly, the diploid number can be reconstructed as 2n=80.

Romania

L. sinapis (RVcoll.07-D500) [2n=74]. This specimen was heterozygous for six chromosomal fusions/fissions. In MI stage 28 bivalents and 6 trivalents were observed. Accordingly, the diploid number can be reconstructed as 2n=28×2+6×3=74.

L. sinapis (RVcoll.06-K557) 2n ca. 68-71. Only mitotic cell divisions were found in this specimen. The diploid chromosome number was counted with an approximation as ca. 68-71.

L. sinapis **(**RVcoll.06-K559) [2n=72]. Only MI cells were found in this specimen with all the metaphase plates demonstrating 36 bivalents. The diploid number can be reconstructed as 2n=72.

L. sinapis (RVcoll.06-K560) 2n=ca. 72-73. Only mitotic cell divisions were found in this specimen. The diploid chromosome number was counted with an approximation as ca. 72-73.

[1] Diploid numbers that were directly counted are given without square brackets.

[2] Diploid numbers that were reconstructed based on MI and MII plates are given in square brackets**.**

L. sinapis (RVcoll.07-F511) 2n=ca. 74. Only mitotic cell divisions were found in this specimen. The diploid chromosome number was counted with an approximation as ca. 74.

L. sinapis (RVcoll.07-F512) 2n=ca. 74. Only mitotic cell divisions were found in this specimen. The diploid chromosome number was counted with an approximation as ca. 74.

L. sinapis (RVcoll.07-D475). Only MI cells were found. The number of bivalents was counted with an approximation as at least 32 or, most likely, more than 32. Accordingly, the diploid number can be reconstructed with an approximation as at least 2n=ca. 64 or, most likely, more than 64.

L. sinapis (RVcoll.06-N005) Izvorul Mure□ului, Harghita, Romania, 870 m 2n= ca.ca. 56 Only mitotic cell divisions were found in this specimen. The diploid chromosome number was counted with an approximation as at least 2n=ca. 56-65 or, most likely, more than 65.

L. sinapis (RVcoll.07-D086) [2n=72]. Only MI cells were found in this specimen with all the metaphase plates demonstrating 36 bivalents. The diploid number can be reconstructed as 2n=72.

L. sinapis (RVcoll.07-D089) [2n=71] Four types of metaphase plates were found in MII cells demonstrating 34, 35, 36 and 37 chromosomes. Most likely, this specimen was heterozygous for three chromosomal fusions/fissions. We did not observe the MI stage in this individual, however, we can reconstruct that the MI cells had 31 bivalents + 3 trivalents (i.e. 2n=71) resulting in different chromosome numbers (n=34, n=35, n=36 and n=37) in the MII cells.

L. sinapis (RVcoll.07-D962) [2n=71]. Two types of metaphase plates were found in MII cells demonstrating 35 and 36 chromosomes. Most likely, this specimen was heterozygous for one chromosomal fusion/fission. We did not observe the MI stage in this individual, however, we can reconstruct that the MI cells had 34 bivalents + 1 trivalents (i.e. 2n=71) resulting in two different chromosome numbers (n=35 and n=36) in the MII cells.

L. sinapis (RVcoll.07-D938) [2n=71]. Two types of metaphase plates were found in MII cells demonstrating 35 and 36 chromosomes. Most likely, this specimen was heterozygous for one chromosomal fusion/fission. We did not observe the MI stage in this individual, however, we can reconstruct that the MI cells had 34 bivalents + 1 trivalents (i.e. 2n=71) resulting in two different chromosome numbers (n=35 and n=36) in the MII cells.

L. sinapis (RVcoll.07-D939) [2n=67]. Two types of metaphase plates were found in MII cells demonstrating 33 and 34 chromosomes. Most likely, this specimen was heterozygous for one chromosomal fusion/fission. We did not observe the MI stage in this individual, however, we can reconstruct that the MI cells had 32 bivalents + 1 trivalents (i.e. 2n=67) resulting in two different chromosome numbers (n=33 and n=34) in the MII cells.

L. sinapis (RVcoll.07-C210) [2n=77]. Two types of metaphase plates were found in MII cells demonstrating 38 and 39 chromosomes. Most likely, this specimen was heterozygous for one chromosomal fusion/fission. We did not observe the MI stage in this individual, however, we can reconstruct that the MI cells had 37 bivalents + 1 trivalents (i.e. 2n=77) resulting in two different chromosome numbers (n=38 and n=39) in the MII cells.

L. sinapis (RVcoll.07-E362) [2n=ca. 72-74]. Only MI cells were found. The number of bivalents was counted with an approximation as ca. 36-37. Accordingly, the diploid number can be reconstructed with an approximation as 2n=ca. 72-74.

L. sinapis (RVcoll.07-E366) [2n=71]. This specimen was heterozygous for three chromosomal fusion/fission. In MI stage 31 bivalents and 3 trivalent were observed. Accordingly, the diploid number can be reconstructed as 2n=31×2+3×3=71.

Romania – summary

Given the karyotype observed in MI and MII cells and taking into account all possible variants of combination of gametes, we conclude that chromosome numbers ranging from 2n=66 to 2n=80 are expected to be found in Romania. In our study we have found (in mitotic cells) or have reconstructed (based on meiotic cells) chromosome numbers from 2n=67 to 2n=77: 2n=67 (1 specimen), 2n=ca. 68-71 (1 specimens), 2n=71 (4 specimens), 2n=72 (2 specimens), 2n=ca. 72-73 (1 specimens), 2n=ca. 72-74 (1 specimens), 2n=74 (1 specimens), 2n=ca. 74 (2 specimens), 2n=77 (1 specimens).

Kazakhstan

L. sinapis **(**RVcoll.06-H631) [2n=56]. Only MII cells were found in this specimen with all the metaphase plates demonstrating 28 chromosomes. The diploid number can be reconstructed as 2n=56.

L. sinapis **(**RVcoll.06-H635) [2n=56]. In MI stage the metaphase plates with 28 bivalents were observed. In MII stage the metaphase plates 28 chromosomes were observed. Accordingly, the diploid number can be reconstructed as 2n=56.

L. sinapis **(**RVcoll.06-H637) [2n=61]. This specimen was heterozygous for one chromosomal fusion/fission. In MI stage the plates with 29 bivalents and 1 trivalent were observed. Accordingly, the diploid number can be reconstructed as 2n=61.

L. sinapis **(**RVcoll.06-H638) [2n=58]. This specimen was heterozygous for two chromosomal fusions/fissions. In MI stage the plates with 26 bivalents and 2 trivalents were observed. Accordingly, the diploid number can be reconstructed as 2n=58.

L. sinapis **(**RVcoll.06-H640) [2n=59]. This specimen was heterozygous for one chromosomal fusion/fission. In MI stage the plates with 28 bivalents and 1 trivalent were observed. Accordingly, the diploid number can be reconstructed as 2n=59.

L. sinapis **(**RVcoll.07-Z210) [2n=62]. Only one MII cell was found in this specimen demonstrating 31 chromosomes. The diploid number can be reconstructed as 2n=62.

L. sinapis **(**RVcoll.07-Z235) 2n=ca. 64. Only mitotic cell divisions were found in this specimen. The diploid chromosome number was counted with an approximation as 2n=ca. 64.

L. sinapis **(**RVcoll.07-Z236) [2n=58]. This specimen was heterozygous for two chromosomal fusions/fissions. In MI stage the plates with 26 bivalents and 2 trivalent were observed. Accordingly, the diploid number can be reconstructed as 2n=58.

L. sinapis **(**RVcoll.07-Z237) 2n=ca. 56. Only mitotic cell divisions were found in this specimen. The diploid chromosome number was counted with an approximation as 2n=ca. 56.

L. sinapis **(**RVcoll.07-Z239) 2n=ca. 64. Only mitotic cell divisions were found in this specimen. The diploid chromosome number was counted with an approximation as 2n=ca. 64.

Kazakhstan – summary

Given the karyotypes observed in MI and MII cells and taking into account all possible variants of combination of gametes, we conclude that chromosome numbers ranging from 2n=56 to 2n=62 are expected to be found in E. Kazakhstan. In our study we have found (in mitotic cells) or have reconstructed (based on meiotic cells) chromosome numbers from 2n=56 to 2n=ca. 64: 2n=56 (3 specimen), 2n=58 (2 specimens), 2n=61 (1 specimens), 2n=62 (1 specimens), 2n=ca. 64 (2 specimens).

Figure S1. Karyotypes of *Leptidea sinapis*. Scale bar corresponds to 10 μm in all figures. Arrows indicate trivalents.

a - Spain, RVcoll.07F568, MI cell demonstrating 53 bivalents; b - Spain, RVcoll.07C470, MII cell demonstrating 53 chromosomes; c - Spain, RVcoll.08H275, 2n=106; d - France, RVcoll.07E253, MI cell demonstrating 49 bivalents; e - France, RVcoll.07E254, MII cell demonstrating 49 chromosomes; f - Italy, RVcoll.07E140, MII cell demonstrating 44 chromosomes; g - Italy, RVcoll.07E140, MI cell demonstrating 43 bivalents; h - Italy, RVcoll.07E140, MII cell demonstrating 43 chromosomes; i - Italy, RVcoll.07E237, MI cell demonstrating 40 bivalents; j - Italy, RVcoll.07E237, MII cell demonstrating 40 chromosomes; k - Romania, RVcoll.06K559, MI cell demonstrating 36 bivalents; l - Romania, RVcoll.07D089, MII cell demonstrating 35 chromosomes; m - Romania, RVcoll.07E366, MI, 31 bivalents and 3 trivalents were observed; n - Romania, RVcoll.07C210, MII cell demonstrating 38 chromosomes; o - Romania, RVcoll.07D938, MII cell demonstrating 36 chromosomes; p - Romania, RVcoll.07F511, 2n=74; q - Kazakhstan, RVcoll.06H631, MII cell demonstrating 28 chromosomes; r - Kazakhstan, RVcoll.06H637, MI cell demonstrating 30 bivalents; s - Kazakhstan, RVcoll.06H638, MI, 26 bivalents and 2 trivalents were observed; t - Kazakhstan, RVcoll.06H640, MI, 28 bivalents and 1 trivalent were observed; u - Kazakhstan, RVcoll.07Z236, MI (intact cell), 26 bivalents and 2 trivalents were observed; v - Kazakhstan, RVcoll.07Z236, squashed MI plate, 26 bivalents and 2 trivalents were observed.

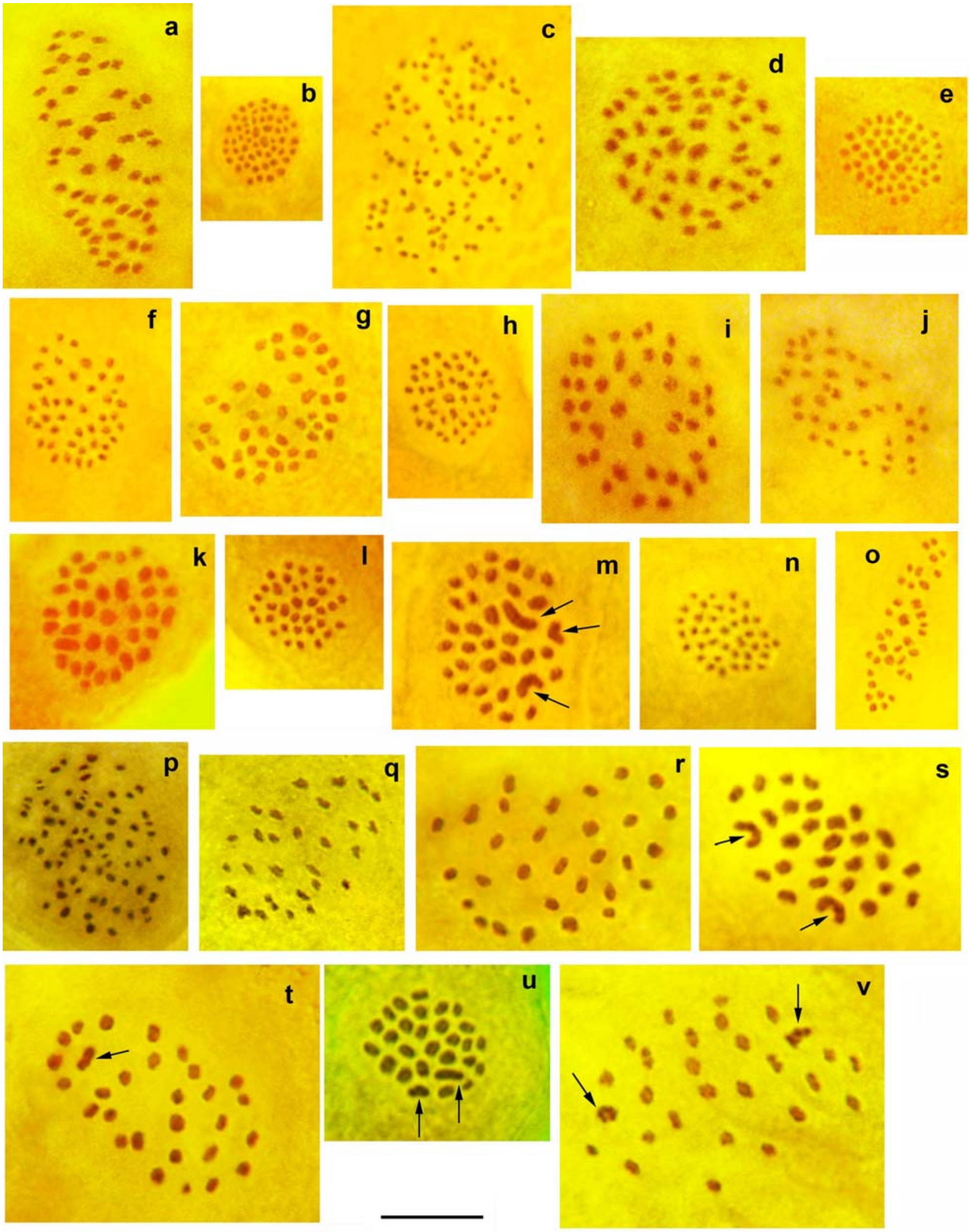
a
b
c
d
e
f
g
h
i
j
k
l
m
n
o
p
q
r
s
t
u
v

Table S1. Discriminant analysis classification results for chromosomal races of *L. sinapis* and *L. reali*. The percentages of correctly identified specimens are shown in bold.

		Category	Predicted group membership						Total
			Spain	France	Italy	Romania	Kazakhstan	*L. reali*	
**Original**	**Count**	Spain	10	0	0	5	4	0	19
		France	1	0	0	4	2	0	7
		Italy	3	0	0	4	4	0	11
		Romania	7	0	0	7	6	0	20
		Kazakhstan	0	0	0	6	10	0	16
		L. reali	0	0	0	0	0	5	5
	**%**	Spain	**52.6**	0	0	26.3	21.1	0	100
		France	14.3	**0**	0	57.1	28.6	0	100
		Italy	27.3	0	**0**	36.4	36.4	0	100
		Romania	35	0	0	**35**	30	0	100
		Kazakhstan	0	0	0	37.5	**62.5**	0	100
		L. reali	0	0	0	0	0	**100**	100
**Cross-validated**	**Count**	Spain	7	0	0	8	4	0	19
		France	1	0	0	4	2	0	7
		Italy	3	0	0	4	4	0	11
		Romania	9	0	0	5	6	0	20
		Kazakhstan	0	0	0	6	10	0	16
		L. reali	0	0	0	0	0	5	5
	**%**	Spain	**36.8**	0	0	42.1	21.1	0	100
		France	14.3	**0**	0	57.1	28.6	0	100
		Italy	27.3	0	**0**	36.4	36.4	0	100
		Romania	45	0	0	**25**	30	0	100
		Kazakhstan	0	0	0	37.5	**62.5**	0	100
		L. reali	0	0	0	0	0	**100**	100

Table S2. List of specimens included in this study. Sample ID, collecting data, GenBank accession codes, as well as *COI* haplotype and chromosome number for specimens that produced results, are shown. Diploid numbers that were directly counted are given without square brackets. Diploid numbers that were established based on MI and MII plates are given in square brackets.

Sample ID	Species	Chromosome number	*COI* haplotype	*COI*	*ITS2*	*CAD*	Genitalia morphometry	Locality	Altitude (m)
RVcoll.08-H275	*L. sinapis*	2n=106	h1	JF512589	JF512813	JF512737	x	Viladrau, Barcelona, Spain	720
RVcoll.08-H277	*L. sinapis*		h1	JF513040			x	Viladrau, Barcelona, Spain	720
RVcoll.08-H278	*L. sinapis*		h2	JF512667			x	Viladrau, Barcelona, Spain	720
RVcoll.08-H281	*L. sinapis*	2n=106	h2	JF512594	JF512814	JF512726	x	Viladrau, Barcelona, Spain	720
RVcoll.08-J393	*L. sinapis*		h1	JF513041			x	Viladrau, Barcelona, Spain	720
RVcoll.09-V341	*L. sinapis*		h1	JF513014			x	Viladrau, Barcelona, Spain	720
RVcoll.09-V342	*L. sinapis*		h1	JF513015			x	Viladrau, Barcelona, Spain	720
RVcoll.09-V343	*L. sinapis*						x	Viladrau, Barcelona, Spain	720
RVcoll.09-V345	*L. sinapis*		h1	JF513016			x	Viladrau, Barcelona, Spain	720
RVcoll.07-C470	*L. sinapis*	[2n=106]	h1	JF512623	JF512808	JF512732	x	Llinars del Vallès, Barcelona, Spain	200
RVcoll.09-V326	*L. sinapis*		h1	JF512590			x	Vallforners, Barcelona, Spain	600-700
RVcoll.09-V327	*L. sinapis*		h1	JF513042			x	Vallforners, Barcelona, Spain	600-700
RVcoll.09-V328	*L. sinapis*		h1	JF513043			x	Vallforners, Barcelona, Spain	600-700
RVcoll.09-V329	*L. sinapis*		h2	JF513044			x	Vallforners, Barcelona, Spain	600-700
RVcoll.09-V330	*L. sinapis*		h1	JF513045			x	Vallforners, Barcelona, Spain	600-700
RVcoll.09-V331	*L. sinapis*		h1	JF513013			x	Vallforners, Barcelona, Spain	600-700
RVcoll.07-C466	*L. sinapis*		h2	JF512663			x	Vallforners, Barcelona, Spain	600-700
RVcoll.07-C467	*L. sinapis*						x	Vallforners, Barcelona, Spain	600-700
RVcoll.07-F568	*L. sinapis*	[2n=106]					x	Vallforners, Barcelona, Spain	600-700
RVcoll.07-E249	*L. sinapis*		h1	JF512585			x	Col de la Chaudière, Drôme, France	1025
RVcoll.07-E250	*L. sinapis*		h3	JF513034			x	NE Bézaudun-sur-Bine, Drôme, France	575
RVcoll.07-E252	*L. sinapis*		h1	JF512586			x	NE Bézaudun-sur-Bine, Drôme, France	735
RVcoll.07-E253	*L. sinapis*	[2n=98]	h1	JF512587	JF512811	JF512747	x	NE Bézaudun-sur-Bine, Drôme, France	735

Sample ID	Species	Chromosome number	*COI* haplotype	*COI*	*ITS2*	*CAD*	Genitalia morphometry	Locality	Altitude (m)
RVcoll.07-E254	*L. sinapis*	[2n=98]	h4	JF512598	JF512812	JF512735	x	NE Bézaudun-sur-Bine, Drôme, France	735
RVcoll.07-E255	*L. sinapis*		h3	JF512599			x	NE Bézaudun-sur-Bine, Drôme, France	735
RVcoll.07-E256	*L. sinapis*		h3	JF512600			x	NE Bézaudun-sur-Bine, Drôme, France	735
RVcoll.07-E138	*L. sinapis*		h1	JF513011			x	Borgo Val di Taro, Parma, Italy	720
RVcoll.07-E140	*L. sinapis*	[2n=87]	h2	JF512593	JF512809	JF512745	x	Borgo Val di Taro, Parma, Italy	620
RVcoll.07-E139	*L. sinapis*		h2	JF513008			x	Passo de Cento Croci, Parma, Italy	1025
RVcoll.07-E141	*L. sinapis*		h2	JF513009			x	Passo de Cento Croci, Parma, Italy	1025
RVcoll.07-E142	*L. sinapis*		h2	JF513038			x	Passo de Cento Croci, Parma, Italy	1025
RVcoll.07-E173	*L. sinapis*		h5	JF513033			x	Ozein-Visyes, Cogne Valley, Italy	1000
RVcoll.07-E174	*L. sinapis*		h5	JF512595			x	Ozein-Visyes, Cogne Valley, Italy	1000
RVcoll.07-E215	*L. sinapis*		h2	JF512665			x	Mompantero Vecchio, Torino, Italy	1340
RVcoll.07-E216	*L. sinapis*		h5	JF512666			x	Mompantero Vecchio, Torino, Italy	1340
RVcoll.07-E217	*L. sinapis*	[2n=ca. 84-86]	h5	JF512596	JF512827	JF512746	x	Urbiano, Mompantero, Torino, Italy	720
RVcoll.07-E237	*L. sinapis*	[2n=80]	h5	JF512597	JF512810	JF512739	x	Novalesa-Moncenisio, Torino, Italy	1155
RVcoll.07-D500	*L. sinapis*	[2n=74]	h1	JF512584	JF512825	JF512730	x	Cheile Babei, Maramureș, Romania	265
RVcoll.06-K557	*L. sinapis*	2n=ca. 68-71	h9	JF513019			x	Bădeni, Cluj, Romania	480
RVcoll.06-K558	*L. sinapis*		h1	JF513036			x	Bădeni, Cluj, Romania	480
RVcoll.06-K559	*L. sinapis*	[2n=72]	h9	JF512580	JF512807	JF512723	x	Bădeni, Cluj, Romania	480
RVcoll.06-K560	*L. sinapis*	2n=ca. 72-73	h9	JF513023			x	Bădeni, Cluj, Romania	480
RVcoll.07-F511	*L. sinapis*	2n=ca. 74	h9	JF513022			x	Bădeni, Cluj, Romania	480
RVcoll.07-F512	*L. sinapis*	2n=ca. 74	h1	JF513031			x	Bădeni, Cluj, Romania	480
RVcoll.07-D475	*L. sinapis*	2n=ca. 64 or, most likely, more than 64.	h1	JF513029			x	Câțcău, Cluj, Romania	255
RVcoll.06-N005	*L. sinapis*	2n= ca. 56-65 or, most likely, more than 65.	h1	JF513018			x	Izvorul Mureșului, Harghita, Romania	870

Sample ID	Species	Chromosome number	*COI* haplotype	*COI*	*ITS2*	*CAD*	Genitalia morphometry	Locality	Altitude (m)
RVcoll.07-D081	*L. sinapis*		h9	JF513037			x	Istrița Hill, Buzău, Romania	350-730
RVcoll.07-D086	*L. sinapis*	[2n=72]	h9	JF513021			x	Istrița Hill, Buzău, Romania	350-730
RVcoll.07-D089	*L. sinapis*	[2n=71]	h1	JF513017			x	Istrița Hill, Buzău, Romania	350-730
RVcoll.07-D962	*L. sinapis*	[2n=71]	h9	JF512581	JF512848	JF512738	x	Valea Mare, Dâmbovița, Romania	225
RVcoll.07-D938	*L. sinapis*	[2n=71]	h10	JF513026			x	Ciupercenii de Olteț, Gorj, Romania	500
RVcoll.07-D939	*L. sinapis*	[2n=67]	h1	JF513010			x	Ciupercenii de Olteț, Gorj, Romania	500
RVcoll.07-D940	*L. sinapis*		h1	JF512662			x	Ciupercenii de Olteț, Gorj, Romania	500
RVcoll.07-C210	*L. sinapis*	[2n=77]	h8	JF512592			x	Buila-Vânturarița Mts., Vâlcea, Romania	750
RVcoll.07-E362	*L. sinapis*	[2n=ca. 72-74	h9	JF512582			x	Pecinișca, Caraș-Severin, Romania	220-320
RVcoll.07-E366	*L. sinapis*	[2n=71]	h9	JF513020			x	Cerna Sat, Caraș-Severin, Romania	525
RVcoll.07-E367	*L. sinapis*		h9	JF513039			x	Cerna Sat, Caraș-Severin, Romania	525
RVcoll.06-H631	*L. sinapis*	[2n=56]	h12	JF513025			x	Landman, Zyryanovsk, Kazakhstan	445
RVcoll.06-H632	*L. sinapis*		h7	JF513047			x	Landman, Zyryanovsk, Kazakhstan	445
RVcoll.06-H633	*L. sinapis*		h9	JF513032			x	Landman, Zyryanovsk, Kazakhstan	445
RVcoll.06-H635	*L. sinapis*	[2n=56]	h9	JF513024			x	Landman, Zyryanovsk, Kazakhstan	445
RVcoll.06-H637	*L. sinapis*	[2n=61]	h11	JF513027			x	Landman, Zyryanovsk, Kazakhstan	445
RVcoll.06-H638	*L. sinapis*	[2n=58]	h9	JF512579	JF512833	JF512729	x	Landman, Zyryanovsk, Kazakhstan	445
RVcoll.06-H640	*L. sinapis*	[2n=59]	h1	JF512583			x	Landman, Zyryanovsk, Kazakhstan	445
RVcoll.06-H641	*L. sinapis*		h5	JF512664			x	Landman, Zyryanovsk, Kazakhstan	445
RVcoll.06-H644	*L. sinapis*		h9	JF513035			x	Landman, Zyryanovsk, Kazakhstan	445
RVcoll.07-Z209	*L. sinapis*		h1	JF513012			x	Saur Mts, Malyi Zhemeney, Kazakhstan	1200-1500
RVcoll.07-Z210	*L. sinapis*	[2n=62]	h11	JF512602	JF512828	JF512748	x	Saur Mts, Malyi Zhemeney, Kazakhstan	1200-1500
RVcoll.07-Z211	*L. sinapis*		h6	JF513046			x	Saur Mts, Malyi Zhemeney, Kazakhstan	1200-1500
RVcoll.07-Z235	*L. sinapis*	2n=ca. 64	h1	JF513030			x	Saur Mts, Malyi Zhemeney, Kazakhstan	1800-2200
RVcoll.07-Z236	*L. sinapis*	[2n=58]	h1	JF512588	JF512829	JF512736	x	Saur Mts, Malyi Zhemeney, Kazakhstan	1800-2200
RVcoll.07-Z237	*L. sinapis*	2n=ca. 56	h6	JF512601			x	Saur Mts, Malyi Zhemeney, Kazakhstan	1800-2200

Sample ID	Species	Chromosome number	*COI* haplotype	*COI*	*ITS2*	*CAD*	Genitalia morphometry	Locality	Altitude (m)
RVcoll.07-Z239	*L. sinapis*	2n=ca. 64	h1	JF513028			x	Saur Mts, Malyi Zhemeney, Kazakhstan	1800-2200
RVcoll.08-M310	*L. reali*			HQ004600			x	Gheorgheni, Harghita, Romania	1000
RVcoll.08-M322	*L. reali*			HQ004596	JF512789	JF512764	x	Gheorgheni, Harghita, Romania	1000
RVcoll.08-M323	*L. reali*			HQ004594			x	Gheorgheni, Harghita, Romania	1000
RVcoll.08-M325	*L. reali*			JF512573	JF512769	JF512760	x	Gheorgheni, Harghita, Romania	1000
RVcoll.07-E553	*L. reali*			HQ004601	JF512767	JF512757	x	Tohanul Nou, Brașov, Romania	700
RVcoll.07-Z083	*L. morsei*			JF512619	JF512837	JF512749		South Altai, Uspenka, Kazakhstan	1460
RVcoll.07-Z124	*L. morsei*			JF512618	JF512839	JF512750		South Altai, Alatai Pass, Kazakhstan	1680

Table S3. Results of morphometric analysis of the male genitalia.

Sample ID	Species	Country	Phallus length (mm)	Saccus length (mm)	Vinculum width (mm)	Phallus / Vinculum	Saccus / Vinculum
RVcoll.07-C470	*L. sinapis*	Spain	1.5	0.56	0.77	1.948	0.727
RVcoll.07-F568	*L. sinapis*	Spain	1.54	0.61	0.78	1.974	0.782
RVcoll.08-H275	*L. sinapis*	Spain	1.75	0.76	0.8	2.188	0.950
RVcoll.08-H281	*L. sinapis*	Spain	1.58	0.59	0.83	1.904	0.711
RVcoll.07-C466	*L. sinapis*	Spain	1.5	0.57	0.74	2.027	0.770
RVcoll.07-C467	*L. sinapis*	Spain	1.59	0.59	0.76	2.092	0.776
RVcoll.08-H277	*L. sinapis*	Spain	1.6	0.64	0.83	1.928	0.771
RVcoll.08-H278	*L. sinapis*	Spain	1.56	0.61	0.78	2.000	0.782
RVcoll.08-J393	*L. sinapis*	Spain	1.61	0.65	0.78	2.064	0.833
RVcoll.09-V326	*L. sinapis*	Spain	1.6	0.63	0.73	2.192	0.863
RVcoll.09-V327	*L. sinapis*	Spain	1.43	0.5	0.71	2.014	0.704
RVcoll.09-V328	*L. sinapis*	Spain	1.6	0.6	0.74	2.162	0.811
RVcoll.09-V329	*L. sinapis*	Spain	1.53	0.54	0.73	2.096	0.740
RVcoll.09-V330	*L. sinapis*	Spain	1.53	0.58	0.72	2.125	0.806
RVcoll.09-V331	*L. sinapis*	Spain	1.71	0.71	0.84	2.036	0.845
RVcoll.09-V341	*L. sinapis*	Spain	1.51	0.59	0.72	2.097	0.819
RVcoll.09-V342	*L. sinapis*	Spain	1.54	0.59	0.74	2.081	0.797
RVcoll.09-V343	*L. sinapis*	Spain	1.51	0.56	0.73	2.068	0.767
RVcoll.09-V345	*L. sinapis*	Spain	1.51	0.55	0.74	2.041	0.743
RVcoll.07-E254	*L. sinapis*	France	1.58	0.59	0.78	2.026	0.756
RVcoll.07-E253	*L. sinapis*	France	1.56	0.6	0.8	1.950	0.750
RVcoll.07-E249	*L. sinapis*	France	1.63	0.65	0.76	2.145	0.855
RVcoll.07-E250	*L. sinapis*	France	1.58	0.65	0.76	2.079	0.855
RVcoll.07-E252	*L. sinapis*	France	1.59	0.63	0.83	1.916	0.759
RVcoll.07-E255	*L. sinapis*	France	1.57	0.66	0.79	1.987	0.835
RVcoll.07-E256	*L. sinapis*	France	1.58	0.67	0.77	2.052	0.870
RVcoll.07-E217	*L. sinapis*	Italy	1.71	0.66	0.78	2.192	0.846
RVcoll.07-E237	*L. sinapis*	Italy	1.53	0.6	0.74	2.068	0.811
RVcoll.07-E140	*L. sinapis*	Italy	1.66	0.67	0.84	1.976	0.798
RVcoll.07-E138	*L. sinapis*	Italy	1.64	0.61	0.82	2.000	0.744
RVcoll.07-E139	*L. sinapis*	Italy	1.6	0.64	0.82	1.951	0.780
RVcoll.07-E141	*L. sinapis*	Italy	1.58	0.55	0.75	2.107	0.733
RVcoll.07-E142	*L. sinapis*	Italy	1.57	0.63	0.77	2.039	0.818
RVcoll.07-E173	*L. sinapis*	Italy	1.5	0.62	0.76	1.974	0.816
RVcoll.07-E174	*L. sinapis*	Italy	1.54	0.6	0.8	1.925	0.750
RVcoll.07-E215	*L. sinapis*	Italy	1.54	0.58	0.78	1.974	0.744
RVcoll.07-E216	*L. sinapis*	Italy	1.63	0.64	0.78	2.090	0.821
RVcoll.07-F511	*L. sinapis*	Romania	1.66	0.59	0.77	2.156	0.766
RVcoll.06-N005	*L. sinapis*	Romania	1.56	0.64	0.82	1.902	0.780
RVcoll.06-K557	*L. sinapis*	Romania	1.54	0.68	0.79	1.949	0.861
RVcoll.06-K560	*L. sinapis*	Romania	1.54	0.63	0.82	1.878	0.768
RVcoll.07-F512	*L. sinapis*	Romania	1.5	0.56	0.75	2.000	0.747
RVcoll.06-K559	*L. sinapis*	Romania	1.65	0.63	0.8	2.063	0.788
RVcoll.07-E366	*L. sinapis*	Romania	1.6	0.59	0.72	2.222	0.819
RVcoll.07-D500	*L. sinapis*	Romania	1.51	0.66	0.75	2.013	0.880
RVcoll.07-D475	*L. sinapis*	Romania	1.44	0.6	0.73	1.973	0.822
RVcoll.07-D939	*L. sinapis*	Romania	1.48	0.59	0.7	2.114	0.843
RVcoll.07-D962	*L. sinapis*	Romania	1.53	0.62	0.76	2.013	0.816
RVcoll.07-D938	*L. sinapis*	Romania	1.58	0.69	0.78	2.026	0.885
RVcoll.07-D086	*L. sinapis*	Romania	1.65	0.66	0.82	2.012	0.805
RVcoll.07-E362	*L. sinapis*	Romania	1.6	0.57	0.73	2.192	0.781

RVcoll.07-D089	*L. sinapis*	Romania	1.59	0.64	0.81	1.963	0.790
RVcoll.07-C210	*L. sinapis*	Romania	1.49	0.59	0.74	2.014	0.797
RVcoll.06-K558	*L. sinapis*	Romania	1.67	0.8	0.81	2.062	0.988
RVcoll.07-D081	*L. sinapis*	Romania	1.6	0.59	0.75	2.133	0.787
RVcoll.07-D940	*L. sinapis*	Romania	1.52	0.58	0.73	2.082	0.795
RVcoll.07-E367	*L. sinapis*	Romania	1.57	0.63	0.75	2.093	0.840
RVcoll.07-Z235	*L. sinapis*	Kazakhstan	1.7	0.65	0.84	2.024	0.774
RVcoll.06-H635	*L. sinapis*	Kazakhstan	1.7	0.7	0.82	2.073	0.854
RVcoll.07-Z236	*L. sinapis*	Kazakhstan	1.6	0.74	0.79	2.025	0.937
RVcoll.06-H637	*L. sinapis*	Kazakhstan	1.58	0.57	0.77	2.052	0.740
RVcoll.07-Z237	*L. sinapis*	Kazakhstan	1.56	0.67	0.8	1.950	0.838
RVcoll.07-Z210	*L. sinapis*	Kazakhstan	1.69	0.71	0.86	1.965	0.826
RVcoll.06-H638	*L. sinapis*	Kazakhstan	1.5	0.62	0.84	1.786	0.738
RVcoll.06-H631	*L. sinapis*	Kazakhstan	1.53	0.58	0.76	2.013	0.763
RVcoll.07-Z239	*L. sinapis*	Kazakhstan	1.75	0.69	0.85	2.059	0.812
RVcoll.06-H640	*L. sinapis*	Kazakhstan	1.59	0.62	0.79	2.013	0.785
RVcoll.06-H632	*L. sinapis*	Kazakhstan	1.61	0.64	0.8	2.013	0.800
RVcoll.06-H633	*L. sinapis*	Kazakhstan	1.55	0.66	0.8	1.938	0.825
RVcoll.06-H641	*L. sinapis*	Kazakhstan	1.62	0.6	0.8	2.025	0.750
RVcoll.06-H644	*L. sinapis*	Kazakhstan	1.58	0.68	0.76	2.079	0.895
RVcoll.07-Z209	*L. sinapis*	Kazakhstan	1.68	0.75	0.84	2.000	0.893
RVcoll.07-Z211	*L. sinapis*	Kazakhstan	1.56	0.63	0.78	2.000	0.808
RVcoll.08-M310	*L. reali*	Romania	2	0.91	0.77	2.597	1.182
RVcoll.08-M322	*L. reali*	Romania	2.02	0.92	0.75	2.693	1.227
RVcoll.08-M323	*L. reali*	Romania	2.06	0.93	0.8	2.575	1.163
RVcoll.08-M325	*L. reali*	Romania	2.02	0.85	0.74	2.730	1.149
RVcoll.07-E553	*L. reali*	Romania	1.96	0.88	0.76	2.579	1.158

Table S4. List of the specimens included in the analysis of geographical longitude vs. chromosome number. In three specimens with different unambiguous chromosome numbers for different cells the mean was used.

**Country**	**Sample ID**	**Longitude (dec. deg.)**	**Chromosome number (n=)**	**log longitude**	**log chromosome number**
Spain	RVcoll.08-H275	2.4	53	0.380211242	1.72427587
Spain	RVcoll.07-F568	2.3	53	0.361727836	1.72427587
Spain	RVcoll.08-H281	2.4	53	0.380211242	1.72427587
Spain	RVcoll.07-C470	2.4	53	0.380211242	1.72427587
France	RVcoll.07-E253	5.2	49	0.716003344	1.69019608
France	RVcoll.07-E254	5.2	49	0.716003344	1.69019608
Italy	RVcoll.07-E140	9.8	43.5	0.991226076	1.638489257
Italy	RVcoll.07-E237	7	40	0.84509804	1.602059991
Romania	RVcoll.06-K559	23.7	36	1.374748346	1.556302501
Romania	RVcoll.07-D086	26.5	36	1.423245874	1.556302501
Romania	RVcoll.07-D938	23.8	35	1.376576957	1.544068044
Romania	RVcoll.07-D089	26.5	35	1.423245874	1.544068044
Romania	RVcoll.07-D962	25.2	35	1.401400541	1.544068044
Romania	RVcoll.07-E366	22.7	34	1.356025857	1.531478917
Romania	RVcoll.07-D939	23.8	33.5	1.376576957	1.525044807
Romania	RVcoll.07-D500	23.4	33.5	1.369215857	1.525044807
Kazakhstan	RVcoll.07-Z210	84.9	31	1.92890769	1.491361694
Kazakhstan	RVcoll.06-H637	84.3	30	1.925827575	1.477121255

Country	Sample ID	Longitude (dec. deg.)	Chromosome number (n=)	log longitude	log chromosome number
Kazakhstan	RVcoll.06-H640	84.3	29	1.925827575	1.462397998
Kazakhstan	RVcoll.06-H631	84.3	28	1.925827575	1.447158031
Kazakhstan	RVcoll.06-H635	84.3	28	1.925827575	1.447158031
Kazakhstan	RVcoll.06-H638	84.3	28	1.925827575	1.447158031
Kazakhstan	RVcoll.07-Z236	84.9	28	1.92890769	1.447158031

Table S5. Estimation of TMRCA of *L. sinapis* under a coalescent model.

Dataset	Median	Mean	Mean StDev	Lower 95% HPD	Upper 95% HPD
COI	30000	41920	5.222E-4	2517	113000
COI (*reali* as outgroup)	29730	42330	1.456E-4	2698	113000
ITS2	30600	42500	5.018E-4	2190	114000
CAD	**30760**	42070	6.719E-4	2274	113000
COI-ITS2-CAD	**8546**	11650	1.609E-4	613.4	31420
COI-ITS2-CAD (*reali* as outgroup)	8825	12970	9.637E-4	677.6	32760

BEAST was used for *COI* (both with and without *L. reali* as outgroup), *ITS2* and *CAD*. *BEAST was used for a multi-locus approach with the three markers (also including and excluding *L. reali* as outgroup). Results were very similar among single markers approaches, but more recent age estimates and a narrower 95% HPD was obtained with the multi-locus analysis. No significant effect of including outgroup was observed. We used the range of medians obtained with the different datasets as an approximation to the TMRCA (maximum and minimum ages in bold). The median is more appropriate than the mean given the LogNormal distribution established as a prior.
